# Disseminated Cutaneous Leishmaniasis and Alcohol Misuse, Northeast Brazil, 2015–2018

**DOI:** 10.3201/eid2709.203714

**Published:** 2021-09

**Authors:** Anastácio Q. Sousa, Pedro D.T. Sindeaux Filho, Diane I.M. Cavalcante, Mércia S. Frutuoso, Francisco F. Pereira, José W.O. Lima, Laécio P.S. Santos, José A.N. Queiroz, James H. Maguire, Richard D. Pearson, Margarida M.L. Pompeu

**Affiliations:** Hospital São José for Infectious Diseases, Fortaleza, Brazil (A.Q. Sousa);; Federal University of Ceará, Fortaleza (A.Q. Sousa, P.D.T. Sindeaux Filho, D.I.M. Cavalcante, M.S. Frutuoso, F.F. Pereira, L.P.S. Santos, J.A.N. Queiroz, M.M.L. Pompeu); S; tate University of Ceará, Fortaleza (J.W.O. Lima);; Harvard University, Boston, Massachusetts, USA (J.H. Maguire);; University of Virginia, Charlottesville, Virginia, USA (R.D. Pearson)

**Keywords:** disseminated cutaneous leishmaniasis, *Leishmania braziliensis*, alcohol, Brazil, leishmaniasis, leishmaniases, alcohol dependence, alcohol use disorder, vector-borne infections, zoonoses, parasitic zoonoses, parasites

## Abstract

Disseminated cutaneous leishmaniasis (DCL) is an uncommon form of *Leishmania braziliensis* infection. It remains unknown why some people develop this clinical condition. We describe 14 DCL patients in Northeast Brazil during 2015–2018. These patients regularly drank large amounts of alcohol, possibly increasing their risk for DCL.

Leishmaniasis is a parasitic disease caused by infection with *Leishmania* parasites, which are transmitted by the bites of phlebotomine sand flies. Localized cutaneous leishmaniasis (LCL), disseminated cutaneous leishmaniasis (DCL), and mucosal leishmaniasis are clinical manifestations of *L. braziliensis* infection. DCL was initially described in the 1980s (*1*,*2*); in 2002, Turetz et al. (*2*) defined DCL as ≥10 cutaneous lesions (papular, nodular, acneiform, crusted, or ulcerated) on ≥2 anatomic regions of the body (i.e., the head, trunk, upper, and lower extremities). *L. guyanensis*, *L. panamensis*, and *L. peruviana* parasites also cause DCL in the New World, whereas *L. tropica* and *L. major* cause DCL in the Old World (*3*). DCL is distinct from anergic diffuse cutaneous leishmaniasis caused by *L. amazonensis*, *L. mexicana*, and *L. aethiopica* infections; anergic diffuse cutaneous leishmaniasis causes multiple nonulcerating, nonhealing lesions in immunocompromised persons (*3*).

In Ceará, a state in Northeast Brazil, only *L. braziliensis* has been isolated from persons who have LCL or DCL (*4*). We observed that many DCL patients in this region report heavy alcohol use. An excessive intake of alcohol can impair the immune response and increase susceptibility to viral and bacterial infections (*5*). Carvalho et al. (*1*) postulated that DCL patients might have a weaker cellular immune response to *Leishmania* spp. than LCL patients. We assessed the association of DCL with heavy alcohol consumption in a region to which *L. braziliensis* is endemic.

## The Study

We conducted the case–control study in an outpatient clinic in the Baturité region, Ceará state, Northeast Brazil, during 2015–2018, when 358 LCL and DCL cases were diagnosed. We identified 18 DCL patients and 38 LCL patients matched by sex, age (within ±5 years), and time of diagnosis. All DCL cases fulfilled the criteria set by Turetz et al. (*2*). Patients with known causes of immunosuppression and pregnant or lactating women were excluded from the study. We collected data on the duration of skin lesions, number and type of lesions, mucosal involvement, underlying conditions (e.g., diabetes, hypertension, etc.) and diagnostic method (i.e., culture, smears, histopathology, or immunohistochemical [IHC] assay). Our histopathological diagnoses were based on inflammatory cell infiltrate patterns and the presence of granulomas and amastigotes. For IHC assays, we used the EnVision FLEX HRP Magenta, High pH (Dako Omnis) kit (Agilent Technologies, https://www.agilent.com) with murine hyperimmune serum from mice infected with *Leishmania braziliensis*. We defined parasite load as the number of intracellular and extracellular amastigotes in 15 high-powered fields (×40) using IHC assays. This work was approved by the Human Ethics Committee of the Federal University of Ceará (Fortaleza, Brazil) (protocol no. 1.552.232 e CAAE 53919816.2.0000.5054).

Participants completed a standardized questionnaire (i.e., the Alcohol Use Disorder Identification Test) to estimate the amount of alcohol intake in grams per day (*6*). We considered ≥28 g/d to be a high level of alcohol consumption (*7*). Most DCL patients were men 19–77 years of age with a duration of disease ranging from 5–36 weeks at diagnosis of leishmaniasis. Each patient had 13–720 lesions on their trunk, limbs, scalp, face, eyelids, conjunctivae, lips, ears, palms, soles of the feet, or genitalia (Figure). Most (56.3%) patients had lesions in the nasal mucosa. Seventeen patients had ≥1 ulcerated lesion; in patient 5, all lesions were ulcerated (Table 1).

**Figure:**
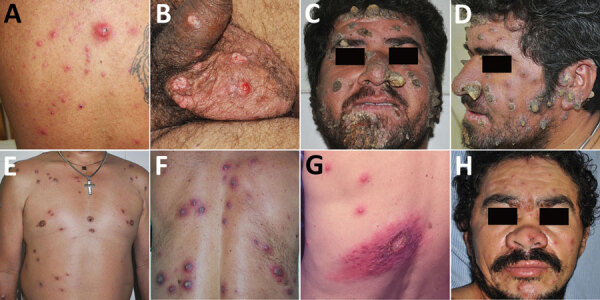
Lesions of patients with disseminated cutaneous leishmaniasis, Baturité region, Ceará State, Northeast Brazil, 2015–2018. Patient numbers match those given in Table 1. A) Ulcerated, acneiform, and papular lesions on the back of patient 1. B) Ulcerated lesions on the genitalia of patient 2. C–D) Crusted and crusted-horny lesions on the face of patient 3. E) Papular, crusted, and ulcerated lesions on the trunk of patient 3. F) Crusted, ulcerated, and papular lesions on the back of patient 6. G) Ulcer surrounded by zosteriform and papular lesions on the back of patient 11. H) Papular, crusted, and ulcerated lesions on the face as well as an ulcerated and crusted-horny lesion on the superior right eyelid of patient 12.

**Table 1:** Clinical, diagnostic and alcohol intake data of 18 patients with disseminated cutaneous leishmaniasis, Baturité region, Ceará State, Northeast Brazil, 2015–2018*

Patient ID	Alcohol intake, g/d	Age, y/sex	Duration of lesions, wks†	No. lesions	Lesion type(s)	Mucosal lesions	Diagnostic method
1	800	25/M	NA	79	U, Ac, P	No	H
2	600	41/M	16	184	Cr, U	Yes	H
3	400	36/M	16	167	U, Cr, crusted-horny, P	Yes	C, H, I
4	400	60/M	5	13	U, P	No	H
5	400	41/M	NA	24	U	NA	H
6	400	49/M	16	171	U, N, Ac, P	No	H, I
7	400	44/M	32	720	U, P, Ac	Yes	H, I
8	300	51/M	36	110	U, N	NA	H
9	240	73/M	24	20	U, Cr, N	No	H
10	230	47/M	24	18	U, Cr, P	No	C, H
11	170	39/M	18	37	P, U, zosteriform	Yes	H
12	140	38/M	6	71	P, Cr, U, crusted-horny	Yes	C, H, I
13	60	19/M	12	14	U, Cr, P	No	H, C
14	45	32/M	32	421	U, P, Cr, N	Yes	C, H
15	0	77/M	32	22	U, N, Ac	Yes	H
16	0	34/F	8	41	U, N	Yes	H
17	0	71/F	8	19	U, P	No	H
18	0	42/M	NA	60	U, N, Cr	Yes	C, H

*Ac, acneiform; Cr, crusted; C, culture; H, histopathology; I, immunohistochemical assay; ID, identification; N, nodular; NA, not available; P, papular; U, ulcerated. †At time of diagnosis.

DCL and LCL patients were well-matched by sex and age (Table 2). DCL patients had longer durations of disease before diagnosis than LCL patients (p<0.01). All LCL lesions were ulcerated and found predominantly in exposed skin areas: lower limbs (50%), upper limbs (25%), head (10%), and trunk (5%). In total, 36 (92%) LCL patients had 1–2 lesions; the other 3 (8%) patients had 3, 5, and 6 lesions. We observed nasal mucosa involvement in only 1 LCL patient.

**Table 2:** Comparison of LCL and DCL patients, Baturité region, Ceará State, Northeast Brazil, 2015–2018*

Variable	Localized cutaneous leishmaniasis	Disseminated cutaneous leishmaniasis	Odds ratio†	p value
Total	38 (100.0)	18 (100.0)		
Sex				
M	35 (92.1)	16 (88.9)	1.00	0.7
F	3 (7.9)	2 (11.1)	1.46	
Age, y‡	41 (19–89)	42 (19–77)	1.01	0.64
Diabetes	3 (7.9)	3 (16.7)	2.13	0.39
Disease duration, wks‡	8 (3–26)	16 (5–36)	1.17	<0.01
Mucosal lesion	1 (2.6)	9 (50.0)	43.7	<0.01
Parasite load‡§	3 (1–340)	5 (1–556)	1.002	0.53
Agricultural occupation	22 (57.9)	12 (66.7)	1.45	0.53
Daily alcohol intake, g/d‡	0 (0–400)	325 (0–800)	1.01	<0.01
Days with alcohol intake >28 g	4 (10.5)	14 (77.8)	23	<0.01

*Values are no. (%), except as indicated. DCL, disseminated cutaneous leishmaniasis; LCL, localized cutaneous leishmaniasis. †Estimated by simple logistic regression. ‡Values are median (range). Load measured as the number of intracellular and extracellular amastigotes in 15 high-powered fields (×40) using immunohistochemical assays. §Of 6 LCL patients and 7 DCL patients.

In total, 14 (78%) DCL patients drank alcohol in the form of cachaça, a popular beverage made by distilling fermented sugar cane juice (*8*). Cachaça has an alcohol content of 40%, similar to that of other distilled spirits such as whiskey, tequila, and vodka. One liter of cachaça or whiskey contains 400 g of pure alcohol. For the 14 patients who drank cachaça, alcohol intake ranged from 45–800 g/d. Twelve (67%) DCL patients drank >350 mL of cachaça (140 g of alcohol) daily. The other 4 (22%) DCL patients did not drink alcohol, including 3 patients who had diabetes. LCL patients had a significantly lower alcohol intake than DCL patients (p<0.01). In total, 25 (64%) LCL patients did not drink alcohol. Fourteen (36%) LCL patients reported alcohol consumption, including 4 who had alcohol intakes >28 g/d, 3 who had intakes of 28–50 g/d, and 1 who had an intake of 400 g/d. In addition, 3 LCL patients had diabetes. We found an association between alcohol intake and parasite load (Spearman ρ = 0.482; p = 0.03).

## Conclusions

The clinical manifestations of DCL in these patients did not differ substantially from those reported previously (*2*,*9*). However, we observed 1 patient who had only ulcerated lesions and another with crusted-horny lesions, both uncommon forms of this rare disease (Figure). The duration of skin lesions before diagnosis was longer in persons with DCL than LCL, similar to the observations of Turetz et al. (*2*). Most DCL lesions were identified by histopathological assays. Our results suggest that DCL is associated with alcohol misuse.

Alcohol causes dysregulation of the innate and adaptive immune responses (*10*). Persons who misuse alcohol have decreased tissue recruitment of neutrophils during bacterial infections and substantial defects in neutrophil function. In addition, these persons have dendritic cells that are fewer in number and have impaired differentiation and function (*11*), possibly causing an imbalance toward a Th2 profile (*12*,*13*). Persons who misuse alcohol produce macrophages with decreased phagocytic and microbicidal activity as well as reduced adherence to other cells in the lesion, which increases their migration to the circulatory system (*5*,*13*). These immune anomalies could explain the correlation between alcohol misuse and parasite load in DCL patients. Vitamin and micronutrient deficiencies are also common in persons who misuse alcohol (*14*) and might also contribute to risk for DCL.

Other risk factors might also contribute to the pathogenesis of DCL. For example, younger age and male sex are associated with DCL (*2*); we controlled for these variables in our analysis. Different strains of *L. braziliensis* might also account for the differential manifestations of LCL and DCL. Cardoso et al. (*15*) showed that neutrophils from healthy persons had decreased microbicidal activity when infected with parasites from DCL patients compared with LCL patients.

In summary, we found an association between DCL and heavy alcohol use. Excessive alcohol intake impairs the human immune system. We postulate that alcohol misuse is a risk factor for DCL in persons infected with *L. braziliensis*. Additional studies are needed to determine whether this association is causal, and if so, to elucidate the mechanism(s) of immune dysregulation responsible for development of DCL in persons infected with *L. braziliensis*. Health officials should consider campaigns focused on preventing sand fly bites in persons who misuse alcohol.
